# Supplementary material for: Control of voluntary and optogenetically perturbed locomotion by spike rate and timing of neurons of the mouse cerebellar nuclei
Source: eLife. 2018 Apr 16;7:e29546. doi: 10.7554/eLife.29546 (PMC5902160; doi:10.7554/eLife.29546)
Supplement: Transparent reporting form [file elife-29546-transrepform.pdf]

eLIFE
1st Floor
24 Hills Road
Cambridge CB2 1JP, UK
P 01223 855340
W elifesciences.org
T @elife

eLife's transparent reporting form

We encourage authors to provide detailed information *within their submission* to facilitate the interpretation and replication of experiments. If you have any questions, please contact us: editorial@elifesciences.org.

Sample-size estimation

- You should state whether an appropriate sample size was computed when the study was being designed
- You should state the statistical method of sample size computation and any required assumptions
- If no explicit power analysis was used, you should describe how you decided what sample (replicate) size (number) to use

Please outline where this information can be found within the submission (e.g., page numbers or figure legends), or explain why this information doesn't apply to your submission:

We expected that we would need recordings from 20-30 cells of each type (Purkinje and cerebellar nuclei), with multiple replications of each stimulus pattern/strength for a range of stimulus patterns/strengths. It was not possible to anticipate the variance and choose specific numbers in advance because analogous work had not been done, because the study entailed examination of the differential responses to apparently identical stimuli and because mice were running ad lib and stimuli could be delivered only as long as mice performed. Therefore, we recorded as many cells as possible per animal and aimed for at least 3 and up to 10 replicates (trials) per stimulus pattern/strength for 9 different patterns/strengths and analyzed the data accordingly. The total number of mice for which data was successfully obtained is given in the first paragraph of the Materials and Methods section. N values for cells and trials are given throughout the manuscript.

Replicates

- You should report how often each experiment was performed
- You should include a definition of biological versus technical replication
- The data obtained should be provided and sufficient information should be provided to indicate the number of independent biological and/or technical replicates
- If you encountered any outliers, you should describe how these were handled
- Criteria for exclusion/inclusion of data should be clearly stated
- High-throughput sequence data should be uploaded before submission, with a private link for reviewers provided (these are available from both GEO and ArrayExpress)

eLife Sciences Publications, Ltd is a limited liability non-profit non-stock corporation incorporated in the State of Delaware, USA, with company number 5030732, and is registered in the UK with company number FC030576 and branch number BR015634 at the address 1st Floor, 24 Hills Road, Cambridge CB2 1JP | August 2014

Please outline where this information can be found within the submission (e.g., page numbers or figure legends), or explain why this information doesn't apply to your submission:

- How often each experiment is performed is included associated with the relevant text.
- We have only biological replication (of animals, cells, and trials, indicated throughout manuscript at relevant points); technical replication is not relevant.
- All N values have been reported in the appropriate places in the text.
- No outliers were excluded from analysis based on their deviations from expected behavior. In the classification of Figure 4, cells whose modulation could not be classified are identified as such and analyzed. As the manuscript explores the range of responses observed, individual data points are included in scatter plots or related graphics.
- All quantitative and qualitative criteria for classification and inclusion of ei ther cells or trials in subsets of analyses are stated in the text as the relevant results are presented.
- High-throughput sequence data: N/A

Statistical reporting

- Statistical analysis methods should be described and justified
- Raw data should be presented in figures whenever informative to do so (typically when N per group is less than 10)
- For each experiment, you should identify the statistical tests used, exact values of N, definitions of center, methods of multiple test correction, and dispersion and precision measures (e.g., mean, median, SD, SEM, confidence intervals; and, for the major substantive results, a measure of effect size (e.g., Pearson's r, Cohen's d)
- Report exact p-values wherever possible alongside the summary statistics and 95% confidence intervals. These should be reported for all key questions and not only when the p-value is less than 0.05.

Please outline where this information can be found within the submission (e.g., page numbers or figure legends), or explain why this information doesn't apply to your submission:

- Statistical methods are given and explained in the last paragraphs of the Materials and Methods. Specifics are also reported for each experiment in the text.
- Raw data (recordings from neurons and paw tracking) are included for each experiment.
- Statistical tests and their relevant parameters are described in the text.
- Exact p values are reported except for $p<0.001$.

(For large datasets, or papers with a very large number of statistical tests, you may upload a single table file with tests, Ns, etc., with reference to page numbers in the manuscript.)

Additional data files ("source data")

- We encourage you to upload relevant additional data files, such as numerical data that are represented as a graph in a figure, or as a summary table
- Where provided, these should be in the most useful format, and they can be uploaded as "Source data" files linked to a main figure or table
- Include model definition files including the full list of parameters used
- Include code used for data analysis (e.g., R, MatLab)
- Avoid stating that data files are "available upon request"

Please indicate the figures or tables for which source data files have been provided:

None.
